# Endocrine system dysfunction and chronic heart failure: a clinical perspective

**DOI:** 10.1007/s12020-021-02912-w

**Published:** 2021-10-28

**Authors:** Giuseppe Lisco, Vito Angelo Giagulli, Michele Iovino, Roberta Zupo, Edoardo Guastamacchia, Giovanni De Pergola, Massimo Iacoviello, Vincenzo Triggiani

**Affiliations:** 1 Interdisciplinary Department of Medicine, Section of Internal Medicine, Geriatrics, Endocrinology and Rare Diseases, University of Bari “Aldo Moro”, School of Medicine, Policlinico, Piazza Giulio Cesare 11, 70124 Bari, Italy; 2 National Institute of Gastroenterology, Saverio de Bellis, Research Hospital, Castellana Grotte, Bari Italy; 3 Clinical Nutrition Unit, Medical Oncology, Department of Internal Medicine and Clinical Oncology, University of Bari, School of Medicine, Policlinico, Piazza Giulio Cesare 11, 70124 Bari, Italy; 4 Department of Medical and Surgical Sciences, Cardiology Department, University of Foggia, Foggia, Italy

**Keywords:** Chronic heart failure, Syndrome of inappropriate antidiuretic hormone secretion, Growth hormone deficiency, Hypothyroidism, Male hypogonadism, Menopause

## Abstract

Chronic heart failure (CHF) leads to an excess of urgent ambulatory visits, recurrent hospital admissions, morbidity, and mortality regardless of medical and non-medical management of the disease. This excess of risk may be attributable, at least in part, to comorbid conditions influencing the development and progression of CHF. In this perspective, the authors examined and described the most common endocrine disorders observed in patients with CHF, particularly in individuals with reduced ejection fraction, aiming to qualify the risks, quantify the epidemiological burden and discuss about the potential role of endocrine treatment. Thyroid dysfunction is commonly observed in patients with CHF, and sometimes it could be the consequence of certain medications (e.g., amiodarone). Male and female hypogonadism may also coexist in this clinical context, contributing to deteriorating the prognosis of these patients. Furthermore, growth hormone deficiency may affect the development of adult myocardium and predispose to CHF. Limited recommendation suggests to screen endocrine disorders in CHF patients, but it could be interesting to evaluate possible endocrine dysfunction in this setting, especially when a high suspicion coexists. Data referring to long-term safety and effectiveness of endocrine treatments in patients with CHF are limited, and their impact on several “hard” endpoints (such as hospital admission, all-cause, and cardiovascular mortality) are still poorly understood.

## Background

Nowadays, around 40 million people live with Chronic Heart Failure (CHF) with a worldwide estimated prevalence of 1–3% in the adult population. Affected patients are usually men, with underlying comorbidities such as coronary artery disease, diabetes mellitus, and arterial hypertension [1]. Both the incidence and prevalence of CHF increase with age. Therefore, as life expectancy is increasing in both genders, the cumulative number of CHF patients and burdens related with CHF chronic complications are expected to increase remarkably over time [2]. To date, around 1 million emergency department and hospital admissions and at least 80,000 deaths per year are attributable to primary acute heart failure, and this overload quadruples when CHF is considered as a comorbidity [3]. It has been estimated that the average per capita cost of CHF accounts for €11,000, mostly (85%) due to recurrent hospital admissions [4].

As another issue, recurring hospital admissions increase the risk of mortality in this cluster of patients, since the mean survival period is 2.6 years after the first hospitalization and progressively decline to 1.8, 1.5, and 1.3 years after the second, third, and fourth hospitalization, respectively [5]. The mortality rate of CHF is elevated in both genders, despite the amelioration of medical and electrical management of the disease. More precisely, 15% of patients die at 30 days after hospital admission, 30% at 1 year, and 65% over 5 years of follow-up [2]. Regardless of etiology [6], CHF progresses through a continuous decline in pump efficiency, myocardial remodeling, and concomitant *sequelae* of adaptive mechanisms [7]. Myocardial remodeling occurs in response to a chronic volume overload and is characterized by myocyte loss, cardiac inflammation and hypertrophy, alteration of extracellular matrix homeostasis, fibrosis, defective autophagy, metabolic abnormalities, and mitochondrial dysfunction [8]. Cardiac output decline activates aortic, carotid, and cardiac baroreceptors with a consequent triggering of different neuroendocrine patterns, including noradrenaline, arginine-vasopressin (AVP), plasma atrial natriuretic peptide (ANP), brain natriuretic peptide (BNP), endothelin, and adenosine release, and activation of the renin-angiotensin-aldosterone system (RAAS) [9, 10]. These initial responses are essential to restore short-term cardiovascular homeostasis, but they become maladaptive [7] in the long-term, also contributing to myocardial remodeling and increasing susceptibility to arrhythmias [11].

### Hormonal disorders in CHF patients

**Fig. 1:**
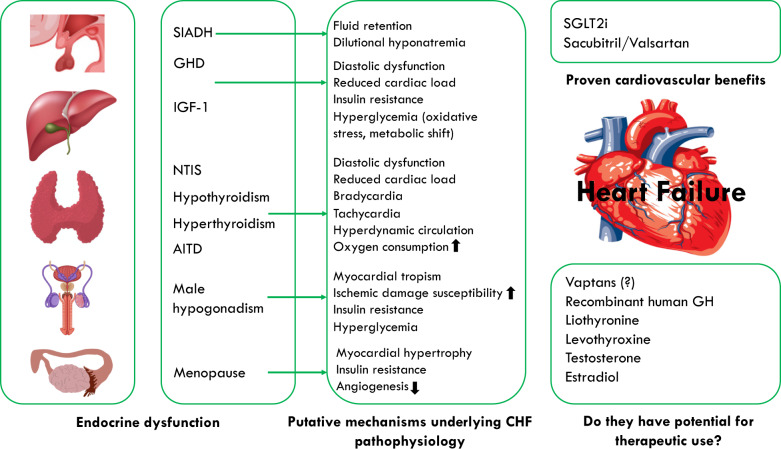
Overview of frequently observed endocrine dysfunction and main mechanisms underlying the CHF pathophysiology. SIADH syndrome of inappropriate antidiuretic hormone secretion, GHD growth hormone deficiency; NTIS non-thyroidal illness syndrome, IGF-1 insulin-like growth factor 1, AITD amiodarone-induced thyroid disorders.

Hormonal disorders have been described in CHF patients (Fig. 1). These include the syndrome of inappropriate antidiuretic hormone secretion, impairment of the growth hormone (GH)—insulin-like growth factor 1 (IGF-1) axis, male hypogonadism, dehydroepiandrosterone sulfate deficiency, and the so-called non-thyroidal illness syndrome (NTIS) [12–14]. These disturbances are particularly evident in CHF patients with reduced ejection fraction [15, 16] even if anabolic hormone deficiencies (HDs) have also been described in patients with preserved ejection fraction [17]. It is unclear whether hormonal changes could be considered purely adaptive mechanisms or, instead, they would deteriorate cardiac pump function, hence suggesting that medical correction of hormonal imbalance could improve the prognosis of CHF. The “Trattamento Ormonale nello Scompenso CArdiaco” (T.O.S.CA.) Registry is a prospective, multicentre, observational study involving 19 Italian centers and evaluating the consequence of endocrine and metabolic disorder in that specific issue. Thyroid hormones, IGF-1, serum T, dehydropianoandrosterone sulfate, insulin resistance, and the presence of diabetes mellitus were evaluated in this setting. Multiple hormones and metabolic deficiencies (MHDs), defined as the presence of at least two HDs, or diabetes mellitus, were diagnosed in 77% of participants. Additionally, MHDs was found to be independently associated with the occurrence of the primary endpoint (cardiovascular mortality and hospital admission due to cardiovascular complications) [HR 1.93 (1.37–2.73), *p* < 0.001] and identified a cluster of individuals with higher mortality risk [HR 2.2 (1.28–3.83), *p* = 0.01]. The more the number of HDs, the more the cumulative risk of worse prognosis [18].

### Natriuretic peptides in CHF

ANP, BNP, and C-type Natriuretic Peptide antagonize both the sympathetic and RAAS hyperactivity, frequently observed in CHF. They promote natriuresis by inhibiting natrium and consequently water reabsorption at the levels of both the proximal and distal renal tubules [19]. In addition, natriuretic peptides induce vasodilatation and reduce myocardial remodeling. Thanks to these mechanisms, natriuretic peptides play a role in preventing volume overload, vasoconstriction, and myocardial injury, especially in the early phases of CHF [20]. Unfortunately, the effectiveness of this adaptive mechanism decreases over time despite the levels of natriuretic peptides continue to raise along with the progression of the disease. This phenomenon is related to decreased availability of biologically active natriuretic peptides (both reduced production and increased enzymatic degradation), increased receptor-mediated clearance, diminished target organ responsiveness (reduced receptor expression, peripheral desensitization, inhibition of downstream signaling), and counter-regulation of the effect of the natriuretic peptides by antagonistic hormonal systems [20].

Besides the diagnostic role of natriuretic peptides, the therapeutic use of these molecules has been extensively evaluated. For example, the recombinant human BNP Nesiritide was found to reduce heart failure symptoms and pulmonary capillary wedge pressure in patients with acute heart failure [21, 22] even if it does not reduce the mortality rate and the frequency of recurrent hospital admissions due to relapsing acute heart failure [23]. Urodilatin, a 32-aminoacid peptide that exhibits similarity to ANP molecular structure [24], is secreted by the distal renal tubules and decreases sodium and water reabsorption at the level of the collecting duct [25, 26]. In a randomized controlled trial, urodilatin reduced right atrial pressure, pulmonary wedge pressure and improved dyspnea severity in patients with symptomatic CHF without any relevant decrease in arterial pressure [27]. Despite these effects, a short-term treatment did not affect clinical composite endpoint nor reduce long-term cardiovascular mortality [28]. Neprilysin is a neutral endopeptidase mainly expressed in the kidneys and degrades NPs and other vasoactive peptides such as angiotensin II, endothelin-1, substance P, and bradykinin.

In the PARADIGM-HF trial, the efficacy of LCZ696 was compared with enalapril in CHF patients with reduced ejection fraction, and the primary end-point was a composite of death due to cardiovascular causes or hospitalization for heart failure [29]. The results showed that patients treated with LCZ696 compared to enalapril had a significant reduction of the primary end-point (21.8% vs. 26.5%, HR 0.80; *p* < 0.001), death for any causes (17.0% vs. 19.8%, HR 0.84; *p* < 0.001), cardiovascular causes (13.3% vs. 16.5%, HR 0.80; *p* < 0.001). Moreover, the risk of hospital admission due to heart failure recurrence was reduced by 21% (*p* < 0.001) [29]. The association valsartan/sacubitril reduced cardiovascular mortality and recurrent hospital admissions in patients with CHF with reduced ejection fraction, but the results in those with preserved ejection fraction appeared uncertain [30]. More specifically, a posthoc analysis of two trials (PARADIGM-HF and PARAGON-HF) found that patients at the highest end of the ejection fraction range had lower overall mortality but higher proportions of noncardiovascular deaths and had not benefited from sacubitril/valsartan compared with angiotensin receptor blockers or angiotensin converting enzyme (ACE) inhibitors alone. Hence, the therapeutic effect of sacubitril/valsartan, compared with angiotensin receptor blockers or ACE inhibitors alone, vary according to the left ventricle ejection fraction (CHF with reduced or mildly reduced ejection fraction) [31, 32].

### Antidiuretic hormone in CHF

AVP is released by the hypothalamic supraoptic nuclei [33, 34] and regulates body water balance [35, 36]. Serum AVP concentrations are elevated in patients with CHF because of a reduction in adequate circulatory blood volume due to decreased cardiac output and arterial pressure [37]. A decline in arterial pressure removes the tonic inhibition of the vagal baroreceptor-mediated afferent pathway, leading to a non-osmotic AVP release [38]. Chronically, this mechanism is responsible for the upregulation of aquaporin-2 (AQP2) expression at distal renal tubules with consequent impairment of water excretion, which worsens the cardiac performance paradoxically due to blood volume expansion [39]. Urinary excretion of AQP2 is directly related to serum AVP concentrations. It is remarkably increased, especially in patients with clinically relevant CHF (New York Heart Association III and IV classes), highlighting its potential role as a biomarker of severe disease in this clinical setting [40]. However, a significant increase in serum AVP levels has also been observed in patients with asymptomatic CHF. Copeptin, a 39-aminoacid glycopeptide that comprises the C-terminal fragment of the AVP precursor, is a stable and sensitive surrogate marker of AVP release. Compared to AVP, copeptin is more stable and less susceptible to pre-analytical variability [41]. Elevated levels of copeptin are associated with all-cause mortality in CHF patients [42, 43], and copeptin was found to be a good predictor of increased 90-day mortality particularly in patients with acute heart failure and high levels of copeptin (quartile 4, cutoff: 57 pmol/L, HR: 3.85 *p* < 0.001) [44]. In a cross-sectional study, copeptin levels were directly correlated with the NT-proBNP levels in patients with reduced ejection fraction, but no correlation between the two biomarkers was found in those with preserved ejection fraction [45]. Hage et al. found that 86 CHF patients with preserved ejection fraction had higher copeptin levels (median 13.56 pmol/L) compared to 62 healthy individuals (5.98 pmol/L; *p* < 0.001). Diastolic dysfunction, assessable in 75 out of 86 patients, was present in 45 of them. However, the levels of copeptin did not correlate with cardiac function but only with NT-proBNP (*r* = 0.223; *p* = 0.04). Prognostic implications of copeptin (i.e., prediction of hospitalization and mortality due to heart failure) were blunted after adjustment for NT-proBNP. In conclusion, copeptin could have a role in a better comprehension of the preserved ejection fraction CHF pathophysiology [46]. However, the role of copeptin is still not clear in clinical practice, and its use in combination with other biomarkers of CHF remains debated [47].

A sustained AVP-dependent antidiuresis in CHF prompts chronic water retention exceeding the RAAS-induced renal natrium reabsorption with a subsequent dilutional (hypervolemic) hyponatremia [39]. Consistent hyponatremia has been observed in more than 20% of hospitalized patients with CHF and was found to be an independent predictor of poor outcomes in this clinical context [48]. The level of serum natrium at admission is inversely correlated with the length of hospital stay, the rate of in-hospital and post-discharge mortality [49], the risk of re-hospitalization due to recurrent heart failure, and worsening CHF events after cardiac resynchronization therapy [50–52]. From a therapeutic point of view, hyponatremia represents a limitation for continuative use of diuretics as they may potentiate maladaptive mechanisms sustaining hyponatremia. The mechanisms by which diuretics worsen the severity of hyponatremia are attributable to impaired renal reabsorption of solutes through a diluting mechanism and inappropriate AVP secretion [53]. Moreover, continuative use of diuretics leads to a remarkable activation of the RAAS. As a consequence, hypokalemia, acid-base disorders, hypomagnesemia, hyperuricemia, lipid disorders, and glucose intolerance [54] may ensue. Furthermore, hyponatremia is a risk factor for the evolution of renal insufficiency during therapy with ACE inhibitors in CHF [55]. On the other hand, long-term use of a non–potassium-sparing diuretic may increase the mortality in CHF patients due to arrhythmic deaths as the consequence of hyperkalemia [56]. Inappropriate secretion of AVP may be rarely induced by amiodarone which is frequently prescribed in patients with CHF and atrial fibrillation, especially in the elderly. The delicate mechanisms involved in it are not entirely understood, but it is thought that amiodarone may induce either hypothalamic AVP release or AQP2 water channel expression at the level of ductal cells apical membrane in the kidney [57, 58].

Finally, the activation of AVP receptor 1 induces systemic vascular contraction, hence activating an additional compensative mechanism by which AVP attempts to raise blood pressure and myocardial afterload [59]. At the myocardial level in neonatal mice, AVP receptor 1 activation was found to induce cardiomyocyte hypertrophy [60]. Vaptans are nonpeptide agents antagonizing the antidiuretic action of AVP by competing with it for binding to V2 receptors in the kidney. Therefore, vaptans increase solute-free water excretion by reducing AQP2 expressions in distal tubules, thus reducing body water content and raising plasma sodium concentration [61]. Vaptans have a rationale in treating chronic hypervolemic and euvolemic hyponatremia as observed in patients with CHF, liver cirrhosis, and inappropriate antidiuretic hormone secretion syndrome [37, 62]. For instance, when used at 15-to-60 mg a day orally, Tolvaptan increased serum natrium levels and reduced fluid retention a few days after the treatment was started [63]. Short- and long-term efficacy is usually scanty, and the use of vaptans does not reduce cardiovascular morbidity and mortality; however, patients reported a relevant improvement of congestive symptoms (e.g., dyspnea, edema, increase in body weight). The results are usually more significant in patients with baseline renal insufficiency [64].

### GH/IGF-1 deficiency in CHF

GH deficiency (GHD) represents one of the most common hormonal deficit observed in patients with CHF. The precise mechanisms explaining this phenomenon remain unclear. Chronic hepatic congestion usually observed in CHF may reduce IGF-1 levels, thus contributing to the decline of the foremost peripheral Growth Hormone (GH) effects. Cittadini et al. found GHD in around 40% of patients with both ischemic and non-ischemic CHF (63 over 158 participants) [65].

GH and IGF-1 reduce peripheral vascular resistence via increasing endothelial synthesis and release of NO and possibly by activating Adenosine Triphosfate sensible potassium channels on smooth muscle cells. GH and IGF-1 may also protect peripheral vasculature from atherosclerosis. Patients with GHD who fail to achieve normal or near-normal IGF-1 levels are prone to develop atherosclerosis and cardiovascular diseases [66]. In an animal study, GH and IGF-1 induced cardiac hypertrophy and increased collagen turnover in the myocardial wall. This piece of evidence is consistent with the fact that GHD patients usually display a reduced ejection fraction, especially during physical exercise, whereas acromegalic individuals are predisposed to myocardial hypertrophy and fibrosis [66].

Patients with CHF are often predisposed to muscular atrophy. Even though several hemodynamic mechanisms may explain this phenomenon, local IGF-1 deficiency has been observed in this cluster of patients. Physical exercise (e.g., 6-month aerobic training) may improve local IGF-1 deficiency without any relevant systemic effects and antagonize muscle atrophy in individuals with moderate CHF [67].

Cittadini et al. recruited 56 patients diagnosed with GHD for a randomized, single-blind, controlled trial (GH replacement versus placebo). CHF severity was classified as follows: 64.4% (18) of participants were diagnosed with class II New York Heart Association (NYHA) heart failure, 21.4% (6) with class III, 14.3% (4) with class IV. GH replacement was associated with a statistically significant improvement in the quality of life, left ventricle ejection fraction, exercise tolerance, and endothelial dysfunction after 6 months of treatment [65]. Similar findings were reported by Napoli et al. after 3 months of GH replacement [68]. As the prevalence rate of GHD among CHF patients may be significantly higher than the general population, it should be considered whether the GHD could be a (con)cause or rather the consequence of CHF (e.g., hypoxic pituitary injury).

Left ventricle function and myocardial remodeling are unlikely to improve after GH replacement therapy in patients with reduced ejection fraction (i.e., <40%) due to coronary artery disease but without GHD [69].

GH therapy may improve left ventricle function, ameliorate resistance to fatigue, and quality of life. However, data are available for a short follow-up (usually 3–6 months). Therefore, it is challenging to confirm GH replacement therapy as a possible medical strategy for improving CHF prognosis over time. In addition, GH administration seems to ameliorate myocardial pump efficiency only in patients with a previously confirmed GHD, not in those without [70]. Nevertheless, a high frequency of GHD among CHF patients is expected. In accordance to current guidelines and recommendations [71, 72], GHD should be suspected in patients with dilated cardiomyopathy with unknown etiology. These patients, therefore, should be tested for GHD and treated if the diagnosis is confirmed.

### Hypothyroidism in CHF

Subclinical and overt hypothyroidism have been frequently observed in patients with CHF [73, 74] and may further impair cardiac pump efficiency.

From a pathophysiological point of view, T3 plays a capital role in regulating the expression of genes involved in myocardial contractility and intracellular calcium handling, and the modifications induced by hypothyroidism or NTIS contribute, at least in part, to decrease pump efficiency in CHF [75, 76]. Chronically uncontrolled hypothyroidism may induce myocardial fibrosis, as thyroid hormones suppress the pro-α1 collagen gene expression. It is associated with a loss in small coronary arteries, thus reducing coronary blood flow [77]. Moreover, patients with hypothyroidism display coronary endothelial dysfunction [78] even if the underlying mechanism is still unclear. In one observational study, Thyroid-Stimulating Hormone (TSH) levels in patients with metabolic syndrome were related directly with circulating endothelin-1s and inversely with nitroxide serum concentration [79], therefore suggesting a possible role of hypothyroidism in inducing directly endothelial dysfunction. On the other hand, patients with hypothyroidism can develop several complications fostering endothelial dysfunction, atherosclerosis, cardiovascular diseases, and CHF, such as baseline systemic inflammation, hyperhomocysteinemia, hypercholesterolemia (LDL and Apolipoprotein B), and insulin-resistance [80, 81]. These results are consistent with the finding of Iervasi reporting a significant increase in the rate of cardiovascular events and mortality, mainly attributable to ischemic diseases, in patients with baseline cardiac diseases and subclinical hypothyroidism compared to euthyroid individuals (hazard ratio 2.40, *p* < 0.02) [82].

Different studies evaluated the prognostic impact of hypothyroidism in patients with CHF. In one study conducted in CHF outpatients on top of the standard care (ACE inhibitors or angiotensin receptor blockers, 93%; beta-blockers, 88%) and 15 months of active follow-up, a slight increase in TSH concentration was independently associated with the occurrence of heart failure progression [83]. Another study involving a more relevant sample of patients confirmed the aforementioned results [84]. In a pooled analysis related to 25,390 cases from six prospective cohorts in the United States and Europe, the risk of acute events related to CHF, such as hospital admission for acute decompensation and death, was higher in subjects with TSH levels included in the range of primary hypothyroidism: hazard ratio 1.01 for TSH 4.5–6.9 mU/L; 1.65 for TSH 7.0–9.9 mU/L; 1.86 for TSH 10.0–19.9 mU/L [85]. In a study by Iacoviello et al., recruiting 762 outpatients with compensated CHF and a median follow-up of 5.1 ± 3.7 years, overt hypothyroidism was independently associated with an increased risk of death and hospital admission due to CHF complications [86]. In a meta-analysis of 13 studies, Ning et al. confirmed that overt and subclinical hypothyroidism were associated to an increased risk of all-cause and cardiac mortality and hospital admission in CHF patients [87].

These results suggest that thyroid replacement therapy aimed to restore euthyroidism in patients with hypothyroidism and CHF should be considered for avoiding poor outcomes [88]. In clinical practice, levothyroxine is more frequently used for restoring euthyroidism where necessary. It is usually started at 12.5–25 mcg with a gradual titration based upon the results of serum TSH levels assessed after around 6 weeks of treatment initiation or adjustments [74]. Long-term levothyroxine treatment in hypothyroid patients was found to be safe, and the achievement of TSH levels between 2 and 2.5 mUI/L may reduce the risk of cardiovascular diseases, including heart failure and all-cause mortality [89]. However, thyroid replacement treatment did not provide benefit nor adverse events among elderly patients with subclinical hypothyroidism [90, 91]. In a Danish retrospective cohort study, levothyroxine replacement did not improve the prognosis of patients with subclinical hypothyroidism and CHF while increasing all-cause mortality, cardiovascular death, and composite cardiovascular events [92, 93]. Thus, these results suggest that, in elderly patients, levothyroxine may lead to detrimental rather than positive effects [94]. However, prospective randomized trials are required to clarify this clinical issue [95, 96].

### Non-thyroidal illness syndrome (NTIS) in CHF

Since triiodothyronine (T3) has a relevant role in myocardial homeostasis, low circulating free T3 (fT3) levels usually observed in CHF may affect cardiovascular prognosis [97]. Patients with the so-called NTIS display typically low circulating levels of fT3 with usually normal TSH and normal or mildly reduced free thyroxine (fT4) values. NTIS could be diagnosed in patients with severe symptomatic systemic diseases, undernourishment, and protein loss [98]. A reduced expression and activity of type 1 5′-monodeiodinase—responsible for the peripheral conversion of T4 in T3—should explain, at least in part, the NTIS. However, as T3 enhances the expression of type 1 5′-monodeiodinase, a decrease of type 1 5′-monodeiodinase expression and activity may also be the consequence rather than the leading cause of the NTIS [99]. Systemic inflammation alters thyroid hormone secretion, as well as metabolism and transport as demonstrated by Bartalena et al. [100]. Interleukin (IL)-1, Tumor Necrosis Factor-alpha (TNF-α), interferon-gamma (IFN-γ), IL-6 may suppress TSH secretion, and may also reduce the synthesis of thyroid hormone-binding protein and type 1 5′-monodeiodinase. In addition, TNF-α and IFN-γ reduce the expression of both thyroglobulin and thyroperoxidase genes in human thyrocytes. The NTIS is a quite rare condition, particularly in mild-to-moderate CHF patients, but it could be observed in around 30% of patients with severe CHF. In the latter case, the NTIS severity directly correlated with poorer prognosis [79]. Thyroid hormones significantly influence cardiovascular homeostasis through genomic and non-genomic effects [101]. Of known, cardiac myocytes do not express an appreciable type 2-deiodinase activity, that is responsible for the intracellular conversion of T4 in T3 upon T4 entry into cytoplasm. Therefore, low circulating levels of T3 reflect directly low T3 concentration into cardiac myocyte cytoplasm with a consequent decline of thyroid hormone effects. Among 281 patients with CHF and a mean follow-up of 7 months, Pingitore found that low T3 circulating levels were an independent predictor of all-cause and cardiac mortality [102]. In one observational study recruiting 111 in patients with dilated cardiomyopathy, 21% of them had abnormalities in thyroid function tests [103]. Moreover, the results showed that the fT3-to-fT4 ratio was significantly correlated with echocardiographic parameters reflecting cardiac remodeling and systolic/diastolic dysfunction [103]. Notably, an fT3-to-fT4 ratio <1.7 was associated with increased mortality risk (sensibility, 100%; specificity, 71%) [103]. Low fT3 concentrations were associated with a higher in-hospital mortality rate (odds ratio 14.4; *p* < 0.001) in patients with acutely decompensated heart failure [104] even if controversial results related to this clinical issue have been published [105]. Hamilton and coll. found that the fT3-to-reverse T3 ratio better predicted worse prognosis (higher frequency of death and cardiac transplantation) among patients hospitalized for advanced cardiac heart failure [106].

Evidence supporting the use of levothyroxine or, more specifically, triiodothyronine supplementation for treating the NTIS in patietns with CHF is currently weak. In most of the cases, clinical trials have been made recriuting a restricted number of participants, most of them admitted to hospital care due to acutely decompensed heart failure, and for a limited number of days or weeks. Despite amelioration in left ventricle kinetic was observed in patients receiving triiodothyronine supplementation intravenously or orally, long-term effictiveness and safety of triiodothyronine supplementation are still unknown [107]. Furthermore, triiodothyronine supplementation in patients with preserved ejection fraction are limited, despite thyroid hormones may improve diastolic dysfunction and possibly having a role in this clinical setting [108].

### Hyperthyroidism in CHF

Development and progression of CHF can also be favored by hyperthyroidism and thyrotoxicosis. Hyperthyroidism, thyrotoxicosis, and CHF are not usually related to each other, differently from how is observed with NTIS. For example, hyperthyroidism may be the consequence of a clinical complication in patients assuming amiodarone [109]. Thyrotoxicosis may be a life-threatening condition characterized by tachycardia, increased myocardial contraction and systolic output, peripheral vasodilatation, augmented tissue thermogenesis, decreased diastolic blood pressure, and renal perfusion with consequent activation of the RAAS [110]. Myocardial energy consumption and oxygen demand increase considerably in this clinical setting, thus predisposing patients to physical exercise intolerance and possibly precipitating cardiovascular events [111]. Over time, patients with uncontrolled hyperthyroidism are prone to develop myocardial hypertrophy with increased cardiomyocyte length and cross-section, left atrial enlargement, and diastolic and systolic dysfunction [112, 113]. These morphological and performing changes are associated with a blunted coronary reserve, and longstanding hyperthyroidism may predispose to myocardial ischemia even in the absence of a baseline coronary artery disease [114]. Moreover, hyperthyroidism may increase the risk of new-onset or recurrent supraventricular arrhythmias, such as atrial fibrillation and flutter [115, 116], and trigger a hypercoagulative activity [92]. The typical hyperdynamic pattern of hyperthyroid patients is attributable to a so-called high-output heart failure [117] in which the cardiac pump efficiency decline is essentially the consequence of a systolic output failure to meet a high peripheral metabolic demand. This clinical condition has been described as a harmful consequence of the so-called thyroid storm [118]. The frequency of preserved or reduced ejection fraction CHF among hyperthyroid patients involves only a minority of cases, usually described in the elderly and among individuals with a long history of uncontrolled hyperthyroidism, as well as in those with pre-existing cardiomyopathies or tachyarrhythmias [119]. However, the incidence rate of CHF is more significant in patients with overt and subclinical hyperthyroidism compared to euthyroid individuals [85]. Hyperthyroid patients with CHF require a prompt restoration of euthyroidism by using anti-thyroid medications or urgent total thyroidectomy to rapidly improve cardiac function, reducing heart rate, oxygen consumption, and cardiac output requirement [120].

### Amiodarone-induced thyroid dysfunction in CHF

Amiodarone-induced thyrotoxicosis (AIT) may be a specific issue among patients with CHF, as the use of amiodarone is more frequently observed in this cluster of patients. AIT has two kinds of typical presentation that may occur at any time of treatment or even months after its discontinuation [121]. Type 1 AIT (AIT 1) usually occurs in patients with a pre-existing thyroid disease within a few months after amiodarone initiation and is related to iodine overload accompanying a chronic assumption of the medication (Job-Basedow mechanism) [122]. Conversely, the onset of type 2 AIT (AIT 2) is typically observed after several months of continuative use of amiodarone in patients without underlying thyroid disease and appears to be related with a direct amiodarone-induced toxicity [123]. However, it is not infrequent that a mixed form of AIT could occur [123]. The management of these forms of hyperthyroidism depends on whether amiodarone withdrawal is feasible and according to the patient’s characteristics (age, underlying comorbidities, type of hyperthyroidism). Generally, AIT 1 does not undergo spontaneous remission, requires amiodarone withdrawal if feasible, and usually responds to thionamides very quickly even if a definitive treatment (both surgical or radioiodine) is usually necessary to achieve a stable remission [123]. Amiodarone discontinuation in patients with AIT 2 is not essential, and the medication can be continued if necessary. Oral glucocorticoids remain the therapy of choice in the case of AIT 2 even if spontaneous remissions have been described in affected patients. In some cases, patients may experience late-onset hypothyroidism as the consequence of a relevant thyroidal damage [124].

Amiodarone can also lead to hypothyroidism [84]. Amiodarone is a source of iodine; it is administered chronically at a maintenance dose of 200–400 mg per day that is expected to induce a relevant iodine overload (6–12 mg per day). This overload may temporarily suppress thyroid hormone biosynthesis because of the complete saturation of intrathyroidal enzymatic systems, a phenomenon otherwise known as the Wolff-Chaikoff effect. The Wolff-Chaikoff effect is dysfunctional in patients with thyroid autoimmunities, such as Hashimoto’s thyroiditis or Graves disease previously treated with thionamides or radioiodine, and could predispose these patients consequently to persistent hypothyroidism after an iodine overload [125]. As another mechanism of action, amiodarone suppresses the activity of both type 1 and type 2 5′-monodeiodinase with a subsequent impairment of T4-to-T3 peripheral conversion and pituitary sensibility to thyroid hormone (transient or persistent increase in TSH circulating levels) [126]. Finally, as additive actions, amiodarone and its primary metabolite (desethylamiodarone) were found to antagonize thyroid hormones entry cell and T3 binding to alpha 1 and beta 1 thyroid hormone receptors into cardiac myocytes. Thanks to these additive mechanisms, amiodarone, leads to a sort of “cardiac hypothyroidism” [127] since it reduces the expression of beta-adrenergic receptors on cardiac myocyte surface, a phenomenon explaining, at least in part, its therapeutic efficacy as anti-arrhythmic and anti-anginal medication [128]. Levothyroxine replacement is the treatment of choice in the case of amiodarone-induced hypothyroidism. However, considering the prolonged clearance and cardiac effects of amiodarone, levothyroxine replacement could be less effective when administered in patients with amiodarone-induced hypothyroidism at the same dose as compared to patients with hypothyroidism due to a different etiology. This assumption is ordinarily well-known in clinical practice and should be emphasized in patients with amiodarone-induced hypothyroidism and CHF since hypothyroidism could be hard to be promptly managed in this clinical context. In conclusion, the role of thyroid function derangement in influencing both the onset and progression of CHF is substantial. For this reason, it is essential to perform a baseline thyroid function check before starting the medication and schedule an appropriate thyroid hormone monitoring to prevent possible thyroid dysfunction upon the treatment has started.

### Male hypogonadism in CHF

Jankowska et al. reported T deficiency among hormonal disorders observed in men with CHF [129]. Male hypogonadism is more prevalent in advancing age and is also known as the Late-Onset Hypogonadism (LOH) [130, 131]. A greater prevalence of T deficiency (also defined as “functional hypogonadism”) has been described in individuals with diabetes mellitus and overweight-obesity syndrome [132] than the general population. CHF is frequently diagnosed in patients appertaining to these categories [133]. Several factors can determine the influence of male hypogonadism in the onset and progression of CHF. Indeed, low total T (TT) serum concentration fosters abdominal fat enlargement [134], insulin resistance, hyperglycemia [135], unfavorable lipid profile, hypertension, and atherosclerosis [136]. Observational studies reported a higher mortality rate, mainly attributable to cardiovascular diseases, among elderly patients with hypogonadism rather than eugonadal men [137–139]. More specifically, male hypogonadism was reported more frequently in men with severe coronary heart disease and CHF [140, 141]. Nevertheless, studies involving younger participants (40–70 years old) failed to confirm the inverse relationship between TT serum concentrations and the mortality rate [142, 143]. A meta-analysis did not show any association between low circulating levels of TT in middle-aged men and cardiovascular risk; thus, it is unclear whether male hypogonadism may prompt detrimental effects on cardiovascular health [144]. A cross-sectional study analyzed the impact of serum TT concentration on left ventricle remodeling and cardiac contractility in middle-aged patients. The results confirmend the aforementioned findings, hence suggesting an overall neutral effect of T deficiency in this cluster of patients [145]. Therefore, it could be speculated that low serum TT levels may be a marker of poor health in men rather than a specific biomarker of cardiovascular risk. However, a mutual negative relationship between male hypogonadism and several risk factors for ischemic cardiovascular disease and CHF have been confirmed [146–148].

From a pathophysiological point of view, T increases muscle strength and resistance as it promotes muscle hypertrophy of both type I or oxidative and type II or glycolytic muscle fibers [149, 150]. Moreover, T enhances the transcription of genes involved in the regulation of muscle growth, such as insulin-like growth factors and the related binding protein-3 and myostatin [151]. Patients with CHF display a reduced muscular tropism, and glycolytic fibers are predominant over type I fibers. These alterations describe a maladaptive condition of skeletal muscle in CHF that may impair physical exercise tolerance leading to fatigue and worse quality of life. Also, loss in muscle fiber number and contractile efficiency increases remarkably the so-called ergoreflex, which is responsible for a further increase in sympathetic activity, heart rate, blood pressure, and cardiac overload in response to muscle work-out [152, 153]. T was found to improve muscle strength and exercise capacity, and the age-related decline in TT serum concentrations is a relevant contributor to an impaired exercise tolerance frequently observed in LOH and elderly men [154, 155]. These data suggest that T could potentially restore muscular efficiency, possibly ameliorating the systemic response to physical exercise and fatigue. In addition, T stimulates cardiomyocyte hypertrophy and protects against cardiotoxicity by modulating both ventricular structure and function [156–158]. T may also reduce the QT interval length, prevent ventricular arrhythmias [159], and modulate the early phase of ventricular repolarization by increasing NO production, K+ channel kinetics, and intracellular calcium concentration [160]. However, in an experimental model of myocardial ischemia-reperfusion injury in adult rats, surgical and pharmacological (flutamide) castrated compared to eugonadal strains displayed lower levels of several indices of myocardial recovery, including cytokines (TNF-α, IL-1β, and IL-6) and apoptotic indices (activation of p38 MAPK, caspase-1, caspase-3, caspase-11, and Bcl-2) [161]. Other authors have also reported similar results in mice [162]. T modulates vascular smooth muscle cells proliferation and protect them against cell senescence, reduces sub-endothelial collagen synthesis, and decreases the expression of adhesion molecules (e.g., VCAM-1) hence supporting vascular integrity and possibly preventing fatty streak formation [163]. In one study recruiting community-dwelling men (>65 years) with limitations in mobility, several chronic comorbidities, and TT < 300 ng/dL, patients were randomized to receive placebo or T replacement therapy (TRT) daily for 6 months. The results showed a relevant amelioration in leg and chest muscle strength, but a greater risk of cardiovascular events was observed among patients treated with T than placebo [164]. Although the number of recruited patients and cardiovascular events were low, the results of this trial suggest that TRT should be used with caution in this cluster of patients.

TRT was found to improve glucose control, also reducing adipose mass in hypogonadal men with diabetes mellitus [165, 166]. T administration in men with mild-to-moderate CHF, regardless of their baseline serum TT levels and aiming to maintain serum T concentrations within the physiologic range, has ameliorated some indices of physical performance, including the peak of oxygen consumption, quadriceps isometric strength, and exercise capacity [167, 168]. Similar data have been observed in older patients (mean age 70 years) and with reduced ejection fraction supplemented with a long-acting intramuscular testosterone preparation.

Among patients with CHF, hypotestosteronemia is not an unusual finding and it may correlate directly to cardiac output. In one observational study, patients with CHF receiving intramuscular T preparations compared to those on placebo (every 2 weeks for 12 weeks) displayed a more significant improvement of physical performance after 12 weeks of treatment [169].

These studies demonstrated that T-related anabolic actions could serve muscle strength in patients with CHF, particularly those with lower baseline serum TT concentration. As poor cardio-respiratory fitness is related to higher mortality rate in patients with CHF [170], it could be speculated that TRT may improve quality of life and reduce the mortality risk among hypogonadal men with CHF [171]. T supplementation may also ameliorate insulin sensitivity in skeletal muscles, leading to greater glucose uptake and utilization, and stimulating endothelial nitric oxide production therefore enhancing vasodilation, blood flow, and oxygen availability during physical exercise [168, 172].

In addition, TRT ameliorates insulin resistance, lipid profile, hyperglycemia that are well-recognized risk factors of atherosclerosis and may affect exercise capacity in patients with CHF [168]. Also, TRT stimulates erythropoietin synthesis, consequently improving red cell mass and myocardial oxygen transport, enhances myocardial contractility and reduces systemic inflammation [163].

On the other hand, data from animal models suggested that T might be implicated in cardiomyocyte hypertrophy, apoptosis, and myocardial fibrosis [173, 174], therefore raising concerns about treating hypogonadal men (TT < 12 nmol/L) with concomitant cardiovascular diseases, mobility limitation, and advanced age [164]. However, there is no evidence of either deleterious or positive effects on the left ventricular function due to T administration, and no significant adverse cardiovascular events were found in CHF patients according to the results of a meta-analysis [171]. It should be noted that only a few patients had severe ventriclular dysfunction (only 2% of patients had IV class NYHA CHF), and most of them did not meet the criteria of frailty and severe chronic comorbidities [171]. Nevertheless, high dose T may increase myocardial collagen deposition, lastly blunting ventricular contractility [175].

Male hypogonadism is considered a predisposing condition favoring worse clinical outcomes in patients with cardiovascular diseases, including CHF, as observed during the ongoing pandemic of Coronavirus disease 2019 or COVID-19 [176, 177]. T replacement in comorbid patients, such as those with obesity and diabetes mellitus, is expected to improve signs and related symptoms of CHF [178–181].

Erectile Dysfunction (ED) can be considered a “sentinel marker” of acute cardiovascular events, especially in men under 65 years or in those with type 2 diabetes mellitus, and may raise the suspicion of cardiovascular complications. ED in CHF may result from pathophysiological and pharmacological treatment, and the medical management of sexual dysfunction may be complicated in this cluster of patients. However, patients with NYHA class I/II and ED receiving type 5 phosphodiesterase inhibitors (vardenafil, tadalafil, and sildenafil) usually exhibit slight adverse events, while cardiopulmonary parameters and quality of life are improved significantly [182] besides an adequate cardiometabolic management [183].

### Estrogen deficiency in CHF

A piece of evidence from both preclinical and clinical studies has highlighted the crucial role of gender in driving cardiac remodeling lastly leading to overt CHF [184]. Myocardial hypertrophy appears to start later in women than men, whereas it is a significant risk factor for CHF severity once established [185]. Of known, premenopausal women show a lower incidence of cardiac diseases than age-matched men, but this protection usually disappears in the postmenopausal phase. Heart failure with preserved ejection fraction is more frequently diagnosed in aging people and, in postmenopausal women, it appears to be related to estrogens depletion [186].

Obese patients display different types of ventricular hypertrophy according to gender. Men usually exhibit concentric cardiac hypertrophy, while women show either concentric or eccentric one. Concentric over eccentric cardiac hypertrophy is considered a relevant risk factor for cardiovascular mortality. These observations may explain the gender difference in obese-related cardiovascular mortality [187]. In addition, circulating natriuretic peptides levels are higher in healthy women (especially in premenopause) than in men and are inversely associated with serum total and free testosterone levels. Given the protective role of these peptides, it could be speculated that baseline endocrine status may influence the efficiency of natriuresis and affect cardiovascular risk more in men than women [188]. Hence, a close relationship between the cardiac and endocrine function and sex steroid hormones milieu may be hypothesized. Several clinical trials indicate that hormone replacement therapy in postmenopausal women reduces cardiovascular risk if it is started within the first years after menopause [189]. Under pathological stimuli such as hypertension, volume overload, and ischemia, sex hormones activate several metabolic pathways facilitating or curbing myocardial hypertrophy. Recent evidence has pointed out the protective role of estrogens in cardiomyopathy pathophysiology. They encompass several metabolic pathways involving angiogenesis and myocardial hypertrophy.

Estrogens exert antihypertrophic effects by binding to their receptors (ERα and ERβ) and attenuating the MEK1/2- ERK1/2-Elk1 signaling cascade. ER stimulation increases intracytoplasmic MKP-1 levels to reduce p38 activation, therefore inducing gene-reprogramming and suppressing hypertrophic response. In addition, ER activation blocks the calcineurin-mediated hypertrophic effect through the NFAT- MEF2/GATA4 mechanism. Moreover, ER induces eNOS-sGCcGMP-PKGIα signaling cascade, which curbs cardiac hypertrophy and facilitates cardioprotection [190, 191]. In addition, estrogens upregulate the transcription of vascular endothelial growth factor, hence playing a role in stimulating neovascularization also in the myocardium [192].

### Diabetes mellitus and insulin resistance in CHF

Diabetes mellitus is a well-recognized risk factor for CHF. The role of insulin resistance and poor glycemic control in the pathophysiology of CHF and therapeutic approaches to manage hyperglycemia have been extensively reviewed elsewhere [193–195]. At least one out of three patients with CHF has diabetes mellitus and this frequency raises to 45% in acutely decompensated patients. Diabetes mellitus decreases remarkably the survival probability in patients diagnosed with CHF, and the risk is strictly affected by diabetes evolution and the level of glucose control over time. Insulin resistance carriers increased risk of heart failure as observed in early stage in patients without evidence of overt cardiac disease [196]. Similar findings have also been found in individuals with apparently uncomplicated type 2 diabetes mellitus whose exercise tolerance was reduced [197]. Insulin resistance is also one of the major contributors of myocardial remodeling in obese patients too [198]. A reciprocal relationship between insulin resistance and heart failure has been described as each one of these conditions may affect the other [199]. Several mechanisms may lead to cardiomyopathy in insulin resistance patients: (1) impaired insulin signaling with consequent reduction of glucose transport (glut 4) and utilization; (2) enanched free fatty acids oxidation with consequent increase of mithocondrial oxidative stress; (3) accumulation of free fatty acids, diacylglycerol and ceramides contributing in the so-called lipotoxicity; (4) adipokines such as tumor necrosis factor alpha, IL-6 and resistine facilitate the proteasomal degradation of IRS1 (through MAPK and mTOR), hence further deteriorating insulin signaling; (5) adipokines such as tumor necrosis factor alpha, IL-6 stimulate the syntesis of angiotensin II and aldosterone which in turn contribute in myocardial oxidative stress [200].

The sodium-glucose cotransporter type 2 inhibitors (SGLT2is), one of the new classes of anti-hyperglycemic medications, hinders renal glucose reabsorption at the level of the proximal tubules and produces a reduction in blood glucose levels in an insulin-independent manner [201]. In addition, SGLT2is reduce the all-cause and cardiovascular mortality in type 2 diabetes patients, also attenuating the risk of first and recurrent hospital admissions or urgent visits due to heart failure regardless of baseline ejection fraction [202–206]. The cardioprotective effects of SGLT2is could be essentially considered a class effect [207] despite different background characteristics of recruited patients in terms of cardiovascular risk and glomerular filtration rate [208]. The positive role of SGLT2is in reducing the risk of recurrent medical consultations or hospital admissions due to heart failure has also been demonstrated in patients without diabetes mellitus and CHF with reduced ejection fraction [209, 210]. The results are attributable to putative extra glycemic effects of SGLT2is that include enhanced osmotic diuresis and natriuresis, plasma volume reduction, improved systolic and diastolic function, and cardiac filling, improved endothelial function, reduced cardiac fibrosis and autophagy, increased circulating levels of erythropoietin and proangiogenic progenitor cells [211, 212].

## Conclusions

Endocrine dysfunction is frequently observed in patients with CHF. It is more prevalent in CHF patients with reduced ejection fraction and most of the evidence is related to this kind of condition. Data about endocrine dysfunction in CHF patients with mildly reduced or preserved ejection fraction are limited but it is thought that it coexists in a smaller percentage. In some cases, endocrine dysfunction may be the consequence of a maladaptive mechanism, and it remains unclear if medical correction of the imbalance is necessary or not (i.e., NTIS in advanced CHF). In other cases, endocrine dysfunction may either coexist or anticipate the CHF onset, also fostering the CHF progression over time. Some guidelines currently recommend to assess thyroid function in all patients with CHF as both hypo- and hyperthyroidism may cause or precipitate CHF [213]. In addition, GHD should be screened (and treated if diagnosed) in patients with dilated cardiomyopathy with unknow etiology and it should be a part of the hormonal screening for etiological assessment of CHF with preserved ejection fraction [71, 72, 214]. No other recommendation is currently available in this field, as data related to other hormonal imbalance and CHF prognosis are still limited. Nevertheless, hormonal assessment, including at least thyroid, gonadal and GH-IGF-1 axes, could be necessary especially if a high clinical suspicion coexists.

As another issue, medical correction of endocrine dysfunction should be made with caution upon the diagnosis has occurred, particularly considering patients’ characteristics (e.g., advanced age), baseline CHF severity, chronic comorbidities, and residual life expectancy. In fact, limited data suggest that endocrine therapies in CHF patients with endocrine dysfunction may improve some of the leading clinical symptoms typically observed in CHF, hence ameliorating the quality of life. Moreover, currently available data are limited to a short-term follow-up and specific clinical settings (i.e., acute decompensated heart failure). Therefore, scanty or null long-term evidence is available considering these treatments as reinforcing therapies to standard chronic care (Table 1) and further well-designed and possibly long-term studies are probably necessary to address this issue.

**Table 1:** Overview of possible therapeutic strategies to correct endocrine dysfunction in CHF patients

Molecules	Underlying endocrine dysfunction	Main effects	Long-term efficacy	Mortality
Vaptans	SIADH [62, 215]	Improvement in QoL [63] Reduction of congestive symptoms [63]	Insufficient [216]	None or insufficient data [216]
rhGH	GHD [217]	Improvement in QoL Amelioration of glucose metabolism Increase in cardiac load Amelioration of exercise tolerance	Insufficient [217]	None or insufficient data [217]
T3	NTIS	Increase in cardiac load [218]	Insufficient [218]	None
LT4	Hypothyroidism [88]	Improvement of endothelial dysfunction Amelioration of glucose metabolism Amelioration of lipid profile Reduction of myocardial fibrosis	More favorable, especially in younger patients [94]	No effect; possible risks in elderly men [92]
Thionamides RAI Thyroidectomy [219]	Hyperthyroidism [110]	Reduction in myocardial oxygen consumption [120] Reduction of RAAS activity [110]	Limited experience	Limited experience
Testosterone	Male hypogonadism	Improvement in QoL Amelioration of glucose metabolism Amelioration of lipid profile Amelioration of exercise tolerance Improvement in left ventricle performance [156–158]	Possible adverse cardiovascular events Fibrosis in case of overexposure to T [164, 175]	None or insufficient data [164]
Estradiol	Menopause/ovarian insufficiency [189]	Reduction of myocardial hypertrophy [190, 191] Improvement in glucose and lipid metabolism Angiogenesis [192]	None	None

*rhGH* recombinant human growth hormone, *T3* liothyronine, *LT4* levothyroxine, *RAI* radioiodine, *SIADH* syndrome of inappropriate antidiuretic hormone secretion, *GHD* growth hormone deficiency, *NTIS* non-thyroidal illness syndrome, *QoL* quality of life, *RAAS* renin-angiotensin-aldosterone system

## Supplementary information

Supplementary Information

## Abbreviations

Amiodarone-induced thyrotoxicosis: AIT
Angiotensin converting enzyme: ACE
Aquaporin-2: AQP2
Arginine-vasopressin: AVP
Atrial natriuretic peptide: ANP
Brain natriuretic peptide: BNP
Chronic heart failure: CHF
Growth Hormone deficiency: GHD
Growth hormone: GH
Hormone deficiencies: HDs
Insulin-like growth factor 1: IGF-1
Interferon-gamma: IFN-γ
Interleukin: IL
Late-Onset Hypogonadism: LOH
Multiple hormones and metabolic deficiencies: MHDs
New York Heart Association: NYHA
Non-thyroidal illness syndrome: NTIS
Renin-angiotensin-aldosterone system: RAAS
Sodium Glucose Cotransporter type 2 inhibitors: SGLT2is
Testosterone: T
Testosterone Replacement Therapy: TRT
Thyroid-stimulating hormone: TSH
Total Testosterone: TT
(free) Thyroxine: (f)T4
(free) Triiodothyronine: (f)T3
Tumor Necrosis Factor-alpha: TNF-α
