# Supplementary material for: Endocrine system dysfunction and chronic heart failure: a clinical perspective
Source: Endocrine. 2021 Oct 28;75(2):360–76. doi: 10.1007/s12020-021-02912-w (PMC8553109; doi:10.1007/s12020-021-02912-w)
Supplement: Supplementary file 1 — Supplementary Information [file 12020_2021_2912_MOESM1_ESM.pdf]

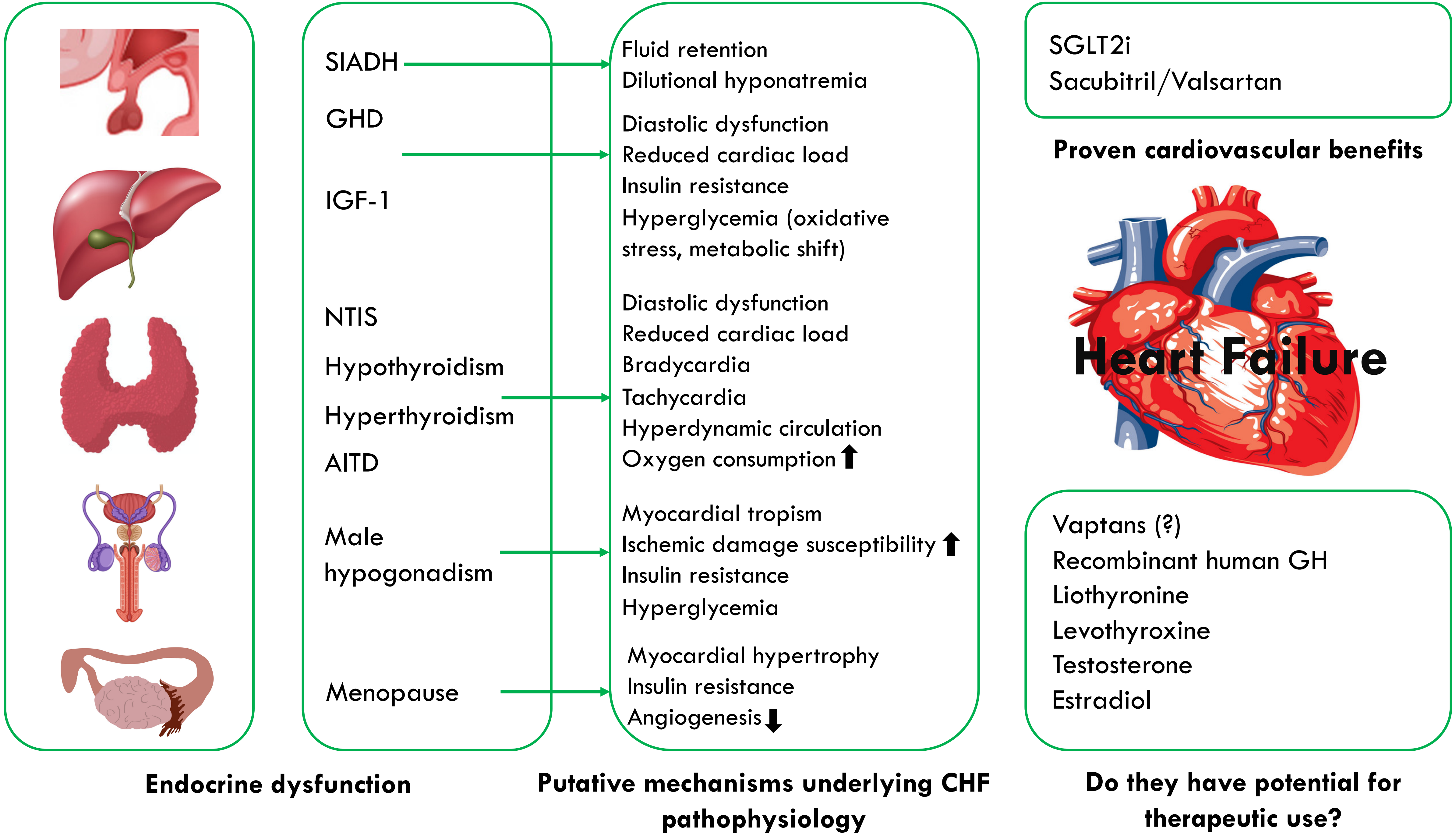
SIADH
GHD
IGF-1
NTIS
Hypothyroidism
Hyperthyroidism
AITD
Male hypogonadism
Menopause
Fluid retention
Dilutional hyponatremia
Diastolic dysfunction
Reduced cardiac load
Insulin resistance
Hyperglycemia (oxidative stress, metabolic shift)
Diastolic dysfunction
Reduced cardiac load
Bradycardia
Tachycardia
Hyperdynamic circulation
Oxygen consumption ⬆
Myocardial tropism
Ischemic damage susceptibility ⬆
Insulin resistance
Hyperglycemia
Myocardial hypertrophy
Insulin resistance
Angiogenesis ⬇
SGLT2i
Sacubitril/Valsartan
Proven cardiovascular benefits
Heart Failure
Vaptans (?)
Recombinant human GH
Liothyronine
Levothyroxine
Testosterone
Estradiol
Endocrine dysfunction
Putative mechanisms underlying CHF pathophysiology
Do they have potential for therapeutic use?
